# *Aspergillus flavus* Promoted the Growth of Soybean and Sunflower Seedlings at Elevated Temperature

**DOI:** 10.1155/2019/1295457

**Published:** 2019-05-02

**Authors:** Ismail, Muhammad Hamayun, Anwar Hussain, Sumera Afzal Khan, Amjad Iqbal, In-Jung Lee

**Affiliations:** ^1^Department of Botany, Abdul Wali Khan University Mardan, Mardan, Pakistan; ^2^Centre of Biotechnology and Microbiology, University of Peshawar, Pakistan; ^3^Department of Agriculture, Abdul Wali Khan University Mardan, Mardan, Pakistan; ^4^School of Applied Biosciences, College of Agriculture and Life Science, Kyungpook National University, Republic of Korea

## Abstract

The current climate changes in the form of global warming are one of the leading threats to agricultural crops (including soybean and sunflower). To enable the crops to cope with the heat stress, innovative steps are needed to be taken as soon as possible. Fungal endophytes are known to secrete secondary metabolites that promote the growth of host plants under stress conditions. Therefore, we have isolated endophytic fungus from* Euphorbia indica* (a wild desert plant) and tested it for plant growth promoting activities. The culture filtrate of the fungal strains exhibited the presence of secondary metabolites. Higher amounts of indole acetic acid (IAA), salicylic acid (SA), flavonoids, and phenolics have been found in the culture filtrate. The 18S rDNA sequence homology and phylogenetic analysis revealed that the isolate is* Aspergillus flavus*. Soybean and sunflower seedlings were inoculated with the identified* A. flavus*. The* A. flavus*-associated seedlings along with the control (without* A. flavus*) were monitored for thermal stress resistance in a growth chamber, operated at 25°C and 40°C. Control seedlings exposed to high temperature stress had higher levels of abscisic acid (ABA), proline, and lower levels of phenols, flavonoids, catalase, and ascorbic acid oxidase. Similarly, a higher reduction in chlorophyll, root-shoot length, and dry weight has been noticed in the control seedlings. The results suggested the usefulness of* A*.* flavus* in host plant growth promotion under heat stress conditions.

## 1. Introduction

In the present era, food shortage is one of the basic problems among the fastest growing population around the world. Man is in a continuous struggle to feed the ever grown population. Climate change on the other hand makes this goal more challenging. Higher levels of temperature, drought, CO_2_, salinity, UV radiations, O_3_, and pathogens are the most common vagaries of climate change that affects the crop quality and yield [1, 2]. Soybean and sunflower are the two important crops cultivated worldwide for the oil and protein. The change in temperature of the growing area poses severe threat regarding the quality yield of these crop species. Having a sessile nature, these crops need some level of manipulability or flexibility in their lifestyle to cope with such changes. Endophytic fungi offer the host plants with great resistance against a variety of biotic and abiotic stresses [3–6]. These endophytes can significantly alter physiological, morphological, anatomical, and molecular aspects of host plants [7–9]. Among these adaptations, phytohormone-balance (ABA, gibberellic acid (GA), IAA, jasmonic acid (JA), and SA), mineral uptake, and enhanced lipids, proteins and carbohydrate contents are noteworthy [10].

Phytohormones are known to be helpful as stress management tools along with the catalases, peroxidases, and ascorbic acid oxidases [11]. IAA, GA, and cytokinin (CK) are categorized as plant growth promoting hormones, while ABA and ethylene are categorized as growth inhibiting hormones [12]. Enhanced proline concentration has also been observed in plant species under different environmental stresses [13]. The accumulation of proline in stressed plant species may or may not be dependent on ABA signaling pathways [13, 14]. The ABA mediated signals regulate the expression of stress related genes. These genes promote the synthesis of osmolytes (proline; polyphenol) to counter the deleterious effect causes by stress [15]. Biotic and abiotic stresses also stimulate the generation of reactive oxygen species (ROS) in plant species [16, 17]. ROS are generally generate in mitochondria and chloroplasts of stressed cells, which are then expand to other parts of plant cells, leading to the programmed cell death (PCD) [18]. Phenolic compounds have the ability to scavenge ROS and reduce oxidative stress in plants undergoing stress. These stresses relieving metabolites (GAs, IAA, ABA, and SA) secreted by the endophytic fungi can play important role in plant growth promotion [19, 20]. The present work has been designed to isolate the endophytic fungi from the wild desert plant* Euphorbia indica* and check its ability in alleviating thermal stress in soybean and sunflower.

## 2. Materials and Methods

### 2.1. Isolation of Endophytic Fungi from* Euphorbia indica*

Endophytic fungi were isolated from the wild plant* Euphorbia indica*, collected from the desert area of District Nowshera Khyber Pakhtunkhwa, Pakistan. The roots and leaves of* Euphorbia indica* were initially washed with tap water and Tween-80 solution. The washed samples were surface sterilized by treating it with 70% ethanol (Sigma Aldrich) for 30 seconds. The samples were then dipped in 5% sodium hypochlorite (sigma Aldrich) for 5 min, followed by 70% ethanol for 30 seconds. The samples were finally washed thrice with double distilled water to remove the traces of ethanol and sodium hypochlorite. The efficiency of the sterilization process was checked by placing some of the uncut samples on Hagam media plates and incubated for 3 days at 28°C. Once sterilized, each root or leave sample was cut into 0.5 cm pieces with sterilized blades [21] and placed (10 pieces/plate) on Hagam media plates (0.5% glucose, 0.05% KH2- PO4, 0.05% MgSO4·7H2O, 0.05% NH4Cl, 0.1% FeCl3, 80 ppm streptomycin and 1.5% agar; pH 5.6 ± 0.2). Streptomycin (80 ppm) was also added to Hagam medium to prevent bacterial growth. The plates were kept in an incubator at 25°C till the emergence of fungal colonies. Pure colonies of fungal strains were obtained on potato dextrose agar (PDA) medium by repeated subculturing. The purified fungal strains were then kept in refrigerator at 4°C till further processing. PDA slants were made to preserve the endophytic fungi for a longer time [22].

### 2.2. Initial Screening of Endophytic Fungi on Rice Seedlings

Rice seedlings at the two leaf stage were used initially to screen the culture filtrates of endophytic fungi for growth promoting or inhibiting activities. Seeds of a commercial rice variety Fakhr-e-Malakand were provided by the Agriculture Research Station, Mingora, Swat, Pakistan. Surface-sterilized rice seeds (sterilized with 5% Clorox for 5 minutes) were incubated at 37°C till the emergence of radical and plumule. Seedlings of uniform sizes were chosen and transferred to flasks (2 seedlings/flask) containing 30 ml of water-agar medium (0.8%). Flasks were then shifted to the growth chamber (day/night cycle: 14 h, 28°C ± 0.3; 10 h, 25°C ± 0.3; relative humidity 70%) for 2 weeks. Fungal strains were grown in Czapek (1% C_6_H_12_O_6_, 1% Peptone, 0.05% MgSO4·7H2O, 0.05% KCl, 0.001% FeSO_4_.7H_2_O; pH 7.3 ± 0.2) broth medium (50 ml) in shaking incubator for 7 days at 120 rpm and 30°C. Fungal biomass was separated from the filtrate using filter paper. Pellets were freeze dried and used for molecular identification of endophytic fungi, while filtrate was used in rice seedlings bioassays. A 100 *µ*l of fungal filtrate was applied to the tip of each seedling at 2 leaf stage. Control treatments (one got distilled water and another got Czapek broth medium instead of fungal filtrates) were also carried out in parallel for the comparison. Growth promoting and inhibiting activities of fungal extracts were recorded by measuring different growth parameters (root-shoot length and dry weight) of rice seedlings after 7 days of incubation [22].

### 2.3. Extraction of DNA from the Isolated Strains

Extraction of DNA from the selected strain was performed, according to well-established protocol of Khan et al., [21]. The extracted DNA was then amplified by PCR. The purity of the extracted DNA and its quantity were measured by Thermo Scientific Nano Drop spectrophotometer at 260 nm [23].

### 2.4. Identification of Fungal Isolate

Selected endophytic fungal strain was identified by amplifying their ITS region of 18 S rDNA with universal primers, ITS-1 (5'-TCC GTA GGT GAA CCT GCGG-3'), and ITS-4 (5'-TCC TCC GCT TAT TGA TAT GC-3') [24]. A 20 ng of gDNA as template was mixed with a 30 *µ*l of EF-Taq (SolGent, Korea) and the mixture was placed in a PCR machine. The conditions of PCR were 95°C for 2 min; 35 cycles (95°C for 1 min, 55°C for 1 min and 72°C for 1 min); 72°C for 10 min. The PCR products along with DNA markers (DNA ladder) were then loaded onto an agarose gel and subjected to electrophoresis for 30 minutes. The gel was developed by using 0.01 g/ml ethidium bromide stain and examined under UV lamp.

### 2.5. Sequencing of Isolated Strains

A purified PCR product of 1600 bp was sequenced with 18S rDNA region by utilizing universal primers ITS-1 (5'-TCC GTA GGT GAA CCT GCGG-3') and ITS-4 (5'-TCC TCC GCT TAT TGA TAT GC-3') [24]. A Big Dye terminator cycle sequencing kit v.3.1 was used for that purpose. Both PCR sequencing and amplification were analyzed by an automated DNA sequencing system (Applied Biosystems, Foster City, USA) at the Macrogen, Inc., Seoul, Korea. The obtained PCR product was initially sequenced and then subjected to a homology search by using online tool, BLAST (https://blast.ncbi.nlm.nih.gov/Blast.cgi).

### 2.6. Determination of IAA and SA in the Culture Filtrates of Isolated Fungal Strain

Culture filtrate of fungal endophyte was screened for the determination and quantification of IAA using Salkowski reagent [25]. Salkowski reagent (2 ml) was mixed with 1 ml of culture filtrate and incubated in the dark at room temperature for 30 minutes. After 30 minutes, the developed color was measured by PerkinElmer Lambda 25 spectrophotometer at 540 nm. A standard curve was constructed by using different concentrations of IAA (11, 20, 30, 40, 60, 80, and 100 *μ*g/ml) and the OD were observed at 540 nm.

Exogenous SA in fugal filtrate was determined by the established protocol of Warrier, Paul, and Vineetha [26]. Fungal hyphae were removed from the culture filtrate by centrifuging it at 10,000g for 10 minutes (Sigma, Model 2-16P centrifuge). The supernatant was collected in a new tube and ice chilled prior to use in the determination of SA. To 100 *μ*l of the supernatant, 2900 *μ*l of freshly prepared ferric chloride (0.1%) was added. OD was taken at 540 nm after the appearance of violet color with the help of spectrophotometer. Standard SA (Sigma) at various concentrations (100, 200, 300, 400, and 500 *μ*g/ml) was used to construct a standard curve.

### 2.7. Symbiotic Association of Isolated Fungal Strain with Soybean and Sunflower

Fungal inoculum was harvested by centrifuging the culture filtrate for 15 minutes at 5000g and 4°C. The harvested inoculum was then grown in Czapek medium (1000 ml water, 300 g potato (sliced, washed), 20 g glucose and 20 g agar; 50 ml in 250 ml conical flask) for 7 days at 30°C. The collected mycelium was then crushed and mixed with water-washed autoclaved sand (1 mg/100 g of sand) in a pot. Seeds of soybean variety Swat-84 and sunflower variety Hysun-33 (9 seeds per treatment) were surface sterilized with sodium hypochlorite and sown in the pot (containing the sand with crushed fungal mycelium). A control experiment was carried out in a similar way, but without the addition of fungal mycelium to the sand. All the pots were kept in a controlled environment (25°C and 40°C) and fed with a half strength Hoagland solution for 2 weeks [27]. The seedlings were harvested after 2 weeks and analyzed for the various growth parameters (including length and dry weight of shoots and roots of seedlings). The experiments were performed in triplicate (each replicate consisted of 9-seedlings).

### 2.8. Estimation of Total Chlorophyll

The total chlorophyll contents of 2-week old soybean and sunflower seedlings were measured with the help of chlorophyll meter (SPAD-502 Minolta, Japan).

### 2.9. ABA Analysis in Sunflower and Soybean

For the analysis of ABA content in soybean and sunflower, Yoon et al. [28] method was applied. Fresh leaves of soybean and sunflower (0.5 g each) were ground in liquid nitrogen. A mixture of isopropanol (1.5 ml) and glacial acetic acid (28.5 ml) was added, filtered, and dehydrated by means of a rotary evaporator. Then diazomethane was added to the mixture and analyzed by GC–MS SIM (6890N network GC system equipped with 5973 network mass selective detector; Agilent Technologies, Palo Alto, CA, USA). Carrier gas was helium at a flow rate of 50 mL/min. The column temperature was programmed to change linearly from 100°C to 250°C at a rate of 10°C per min during 15 min and then stay at 250°C for 5 min. Samples (2-*µ*1) in triplicate were injected using the splitless injection mode. The Lab-Base (Thermo Quset, Manchester, UK) data system software was used to observe responses to ions with m/z values of 162 and 190 for Me-ABA and 166 and 194 for Me-[2H6]-ABA. ABA ([2H6]-ABA) (Sigma Aldrich) was used as internal standard.

### 2.10. Analysis of Antioxidants in Soybean and Sunflower Seedlings

Luck [29] protocol was used for the analysis of CAT concentration in soybean and sunflower seedlings. Fresh leaves (2 g) of soybean and sunflower was crushed in phosphate buffer (10 ml) and centrifuged for five minutes at 10,000g. H_2_O_2_-phosphate buffer (3 ml) and supernatant (40 *µ*l) were mixed and the OD was taken at 240 nm. H_2_O_2_-free phosphate buffer solution was used as blank. One enzyme unit was calculated as the amount of enzyme required to decrease the absorbance at 240 nm by 0.05. Oberbacher and Vines [30] protocol was applied for the estimation of AAO concentration in soybean and sunflower seedlings. Fresh leaves (0.1 g) of soybean and sunflower were ground in phosphate buffer (2 ml) and centrifuged for five minutes at 3000g. Approximately, 3 ml of the substrate solution (8.8 mg of ascorbic acid in 300 ml phosphate buffer, pH 5.6) was mixed with 100 *µ*l of supernatant. The OD_265_ was finally observed at an interval of 30 seconds for 5 minutes. One unit of AAO was calculated by a decrease in OD_265_ of 0.05 per minute.

### 2.11. Analysis of Phenolics, Proline, and Flavonoids in the Culture Filtrate of Endophytic Fungi, Sunflower, and Soybean

Cai et al. [31] procedure was applied for the analysis of phenolics in soybean, sunflower, and fungal filtrate. Different concentrations (100, 200, 300, 500, and 600, 700 and 900 mg/ml) of Gallic acid (Sigma Aldrich) were used to make a standard curve. For the determination of proline, we used the method of Bates et al. [32] with some modifications. A standard curve was plotted by using different concentrations (2, 4, 6, 8 and 10 *µ*g/ml) of proline (Sigma Aldrich). The OD was recorded at 520 nm. Mervat et al. [33] method was applied for the analysis of total flavonoids in soybean, sunflower and fungal filtrate. A standard curve was made by using different concentrations of quercetin (15, 30, 60, 120, 240 and 480 *μ*g/ml, Sigma Aldrich) and the OD was measured at 415 nm.

### 2.12. Analysis of Total Proteins, Lipids, and Soluble Sugars in Soybean and Sunflower Seedlings

The method of Lowry et al. [34] was applied for the analysis of total proteins in the seedlings of soybean and sunflower. A standard curve was made using different concentrations (20, 40, 60, 80, and 100 *µ*g/ml) of BSA (Sigma Aldrich) and the OD was taken at 650 nm. For the determination of total lipids, we used the method of Van Handel [35] with some modifications. A standard curve was made using different concentrations of canola oil (10, 40, 70, 100, 130, and 160 *µ*g/ml) and the OD was recorded at 490 nm. For the analysis of total soluble sugars in the soybean and sunflower seedlings, Mohammadkhani and Heidari [36] method was used. A standard curve was made using different concentration (20, 40, 60, 80, and 100 *µ*g/ml) of glucose (Sigma Aldrich) and the OD was measured at 485 nm.

### 2.13. Statistical Analysis

All experiments were performed 3 times. ANOVA (one way analysis of variance) was used for data analysis and means were compared by Tukey HSD test at p < 0.05 using SPSS-20 (SPSS Inch., Chicago, IL, USA) for windows.

## 3. Results

### 3.1. Isolation of EuR-6 and Its Initial Screening on Rice Seedlings

**Figure 1:**
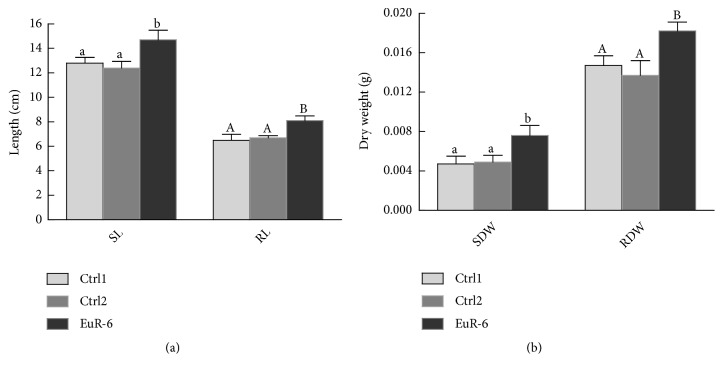
Effect of EuR-6 culture filtrate on the growth of rice seedlings. Ctrl1 represents distilled water; Ctrl2 represents Czapek broth medium; EuR-6 represents* A. flavus*. (a) represents shoot and root lengths of rice seedlings with or without* A. flavus* association; SL = shoot length and RL = root length. (b) represents shoot and root dry weights of rice seedlings with or without* A. flavus* association; SDW = shoot dry weight and RDW = root dry weight. Data are means of 3 replicates (each replicate consisted of 9-seedlings) with standard error. For each set of treatment, the different letter indicates significant differences at P = 0.05 as estimated by Duncan's Multiple Range Test.

EuR-6 was isolated from the roots and leaves of* Euphorbia indica* (Figure S1). Initially, Eur-6 was isolated on Hagam minimal medium and then purified on PDA media plates on the basis of morphological differences. The isolated strain Eur-6 was screened for its growth promoting or inhibiting activity (Figure S2). The culture filtrate (100 *µ*L) was applied on the tip of rice seedlings (grown in 0.8% water-agar medium) at the two leaf stage. Growth parameters of rice seedlings were recorded after one week of filtrate application. A control experiment was carried out side by side for the comparison. The results revealed that the endophytic strain EuR-6 has promoted the rice growth and selected for further experiments (Figure 1).

### 3.2. Identification and Phylogenetic Analysis of Isolated Fungal Strain EuR-6

**Figure 2:**
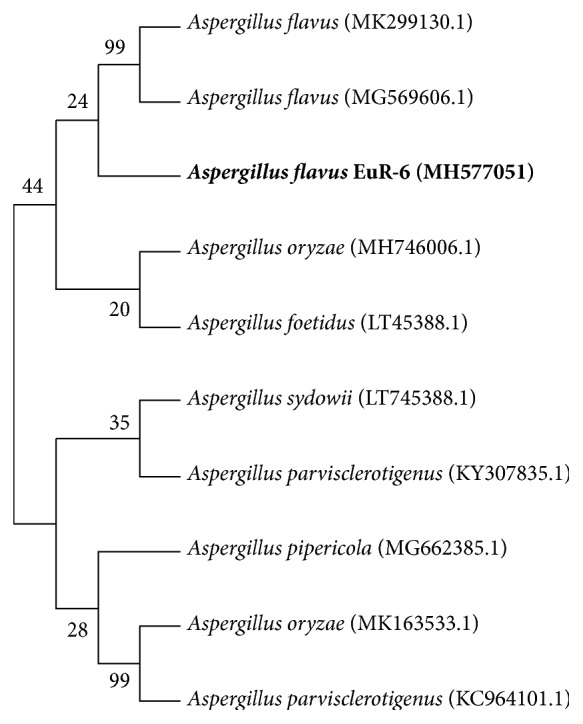
Molecular Identification of plant growth promoting endophyte EuR-6. The evolutionary relationships of EuR-6 were computed using the maximum parsimony (MP) method. The strain EuR-6 was identified as* A. flavus*.

The fungal endophyte EuR-6 has been identified through sequencing of ITS (internal transcribed spacer) region of 18S rDNA. The fungal isolate has been identified by relating ITS region of the EuR-6 with the associated sequences existing in the GenBank database of NCBI (http://www.ncbi.nlm.nih.gov/BLAST/). BLAST result of 18S rDNA sequencing was analyzed via MEGA 7.0 software. Results of BLAST search showed highest sequence similarity with* Aspergillus flavus*, having 99% sequence homology in the MP Dendrogram. The isolated fungus EuR-6 has been identified as* A. flavus* through phylogenetic analysis and sequence homology (Figure 2). The sequence was submitted to the gene bank under accession number MH577051.

### 3.3. Determination of Secondary Metabolites in* A. flavus*

**Figure 3:**
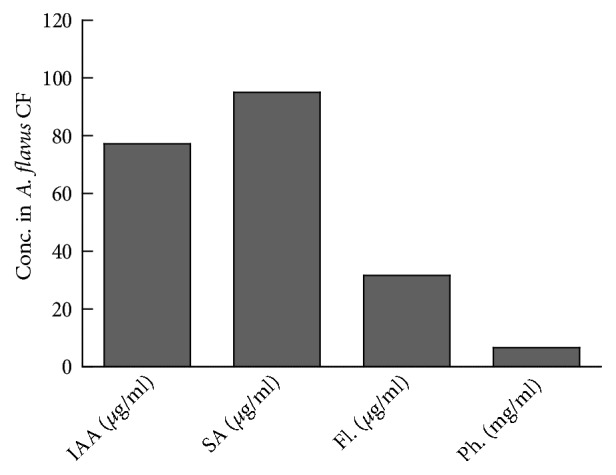
Secondary metabolites in the culture filtrate of* A. flavus*. Conc. = concentration; CF = culture filtrate; SA = salicylic acid; Fl. = flavonoids; Ph. = phenolics.* A. flavus* was grown in Czapek broth medium at 120 rpm for 7 days at 28°C in shaking incubator. The culture filtrate was collected through filtration and tested for the presence of various metabolites.

Culture filtrate (CF) of* A. flavus* contained optimum amounts of growth promoting secondary metabolites (Figure 3). The concentration of IAA in the culture filtrate of* A. flavus* was 77.26 *µ*g/ml, whereas the concentration of SA was 95.0 *µ*g/ml. Similarly, appreciable quantities of flavonoids (31.64 *µ*g/ml) and phenols (6.63 mg/ml) were found in the CF of* A. flavus* (Figure 3).

### 3.4. Role of* A. flavus* on the Growth of Soybean and Sunflower Seedlings Under High Temperature

**Figure 4:**
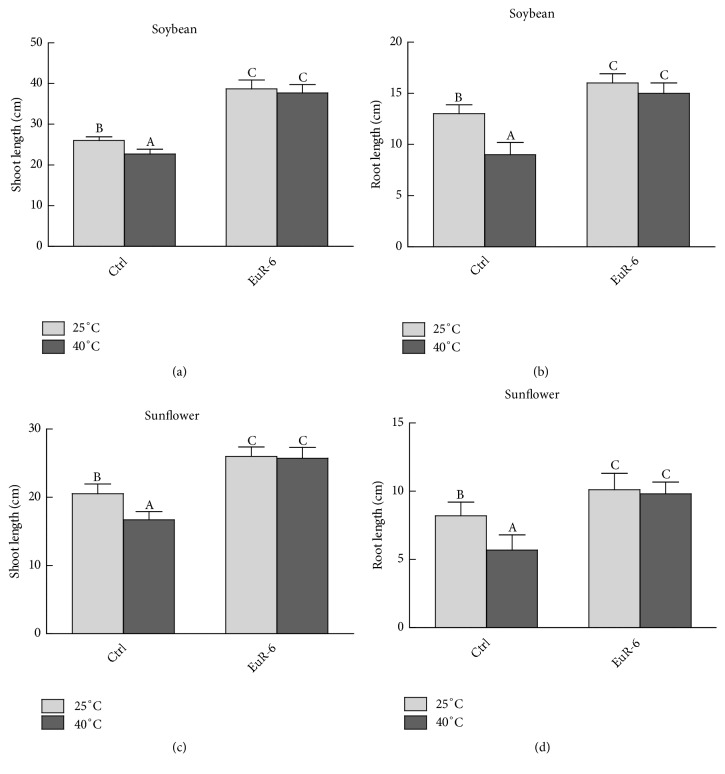
Effect of* A. flavus* on shoot and root lengths of soybean and sunflower seedlings. (a) represents the shoot length of soybean seedlings at 25°C and 40°C; (b) represents root length of soybean seedlings at 25°C and 40°C; (c) represents shoot lengths of sunflower seedlings at 25°C and 40°C; (d) represents root lengths of sunflower seedlings at 25°C and 40°C; Ctrl =* A. flavus*-free seedlings; EuR-6 =* A. flavus*-associated seedlings. Data are means of 3 replicates with standard error. Different litters are significantly different (p < 0.05) as estimated by Duncan's Multiple Range Test.

**Figure 5:**
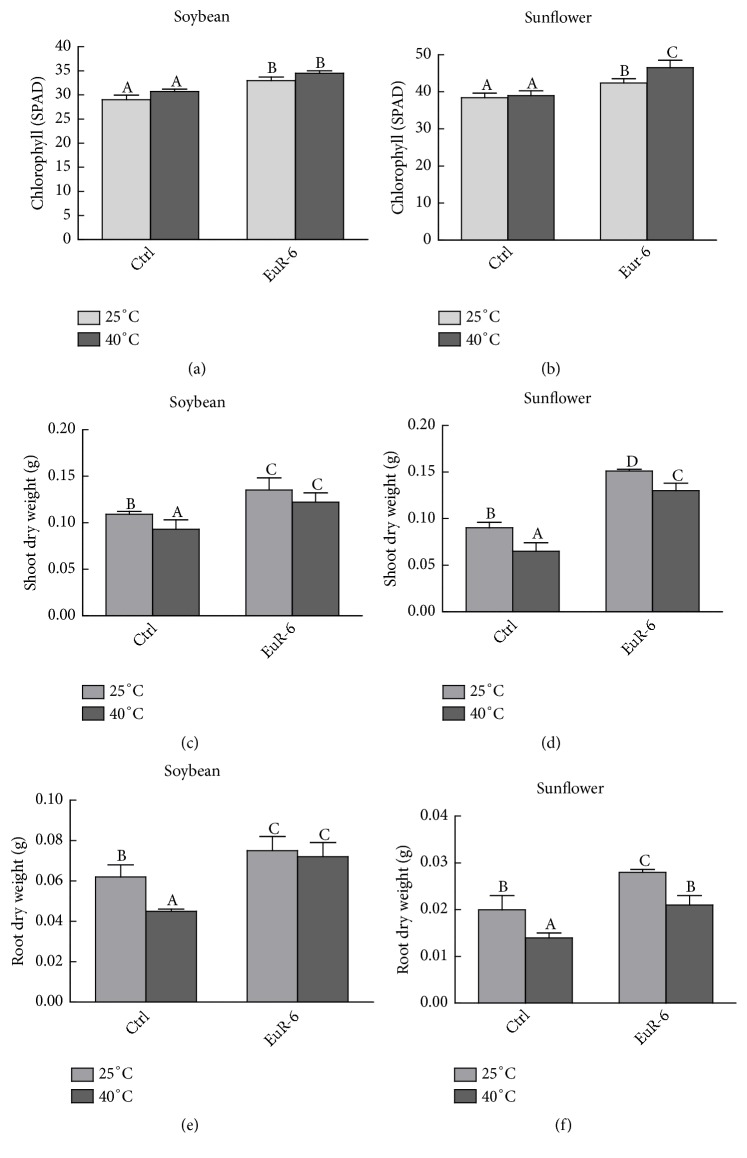
Effect of* A. flavus* on chlorophyll, shoot and root weights of soybean and sunflower seedlings. (a) represents the total chlorophyll contents of soybean seedlings at 25°C and 40°C; (b) represents total chlorophyll contents of sunflower seedlings at 25°C and 40°C; (c) represents shoot dry weight of soybean seedlings at 25°C and 40°C; (d) represents shoot dry weight of sunflower seedlings at 25°C and 40°C; (e) represents root dry weight of soybean seedlings at 25°C and 40°C; (f) represents root dry weight of sunflower seedlings at 25°C and 40°C; Ctrl =* A. flavus*-free seedlings; EuR-6 =* A. flavus*-associated seedlings. Data are means of 3 replicates with standard error. Different litters are significantly different (p < 0.05) as estimated by Duncan's Multiple Range Test.

Significant improvement in the growth of soybean and sunflower seedlings has been recorded, when inoculated with* A. flavus* mycelium. Seedlings of soybean and sunflower cocultured with* A. flavus* displayed significantly (P = 0.05) higher shoot and root growth as compared to the control seedlings under 25°C and 40°C of heat treatments (Figure 4). Total chlorophyll contents of soybean and sunflower seedlings from the control treatment were significantly (P = 0.05) lower as compared to the* A. flavus*-associated seedlings at 25°C or 40°C (Figures 5(a) and 5(b)). Moreover, a significant (P = 0.05) increase in shoot and root dry weights of soybean and sunflower has been recorded in* A. flavus* inoculated plants compared to noninoculated plants grown at 25°C or 40°C (Figures 5(c), 5(d), 5(e), and 5(f)).

### 3.5. Alteration in Endogenous ABA and Proline Contents of* A. flavus*-Associated Soybean and Sunflower Seedlings

**Figure 6:**
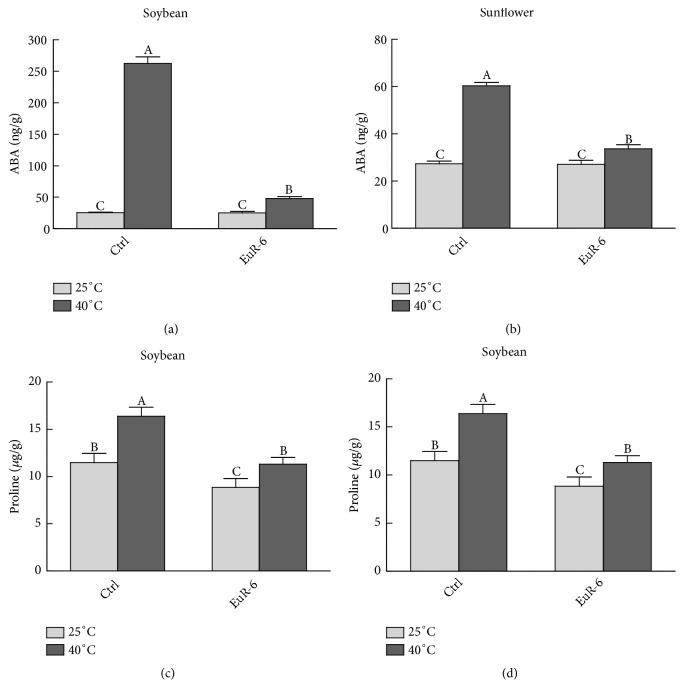
Effect of* A. flavus* on ABA and proline contents of soybean and sunflower seedlings. (a) represents the ABA contents of soybean seedlings at 25°C and 40°C; (b) represents ABA contents of sunflower seedlings at 25°C and 40°C; (c) represents proline contents of soybean seedlings at 25°C and 40°C; (d) represents proline contents of sunflower seedlings at 25°C and 40°C; Ctrl =* A. flavus*-free seedlings; EuR-6 =* A. flavus*-associated seedlings. Data are means of 3 replicates with standard error. Different litters are significantly different (p < 0.05) as estimated by Duncan's Multiple Range Test.

Soybean seedlings without* A. flavus* association and treated at 40°C had accumulated significantly higher (P = 0.05) amounts of ABA (Figure 6(a)). The accumulated amount of ABA in nonassociated seedlings was almost 5.5 times higher than the* A. flavus*-associated soybean seedlings germinated at 40°C. However, a nonsignificant (P=0.05) change was observed in inoculated and noninoculated soybean seedlings maintained at 25°C (Figure 6(a)). Similarly, in sunflower both the control and* A. flavus* inoculated seedlings have almost the same level of ABA at 25°C. However, at 40°C the control seedlings had higher quantities of ABA (60.3 ng/g) as compared to the inoculated seedlings (33.6 ng/g) (Figure 6(b)). Moreover,* A. flavus* noninoculated soybean and sunflower seedlings kept at 25°C or 40°C showed significant (P = 0.05) changes in the proline contents as compared to the* A. flavus* inoculated seedlings (Figures 6(c) and 6(d)).

### 3.6. Total Endogenous Phenolic and Flavonoid Contents of Soybean and Sunflower

**Figure 7:**
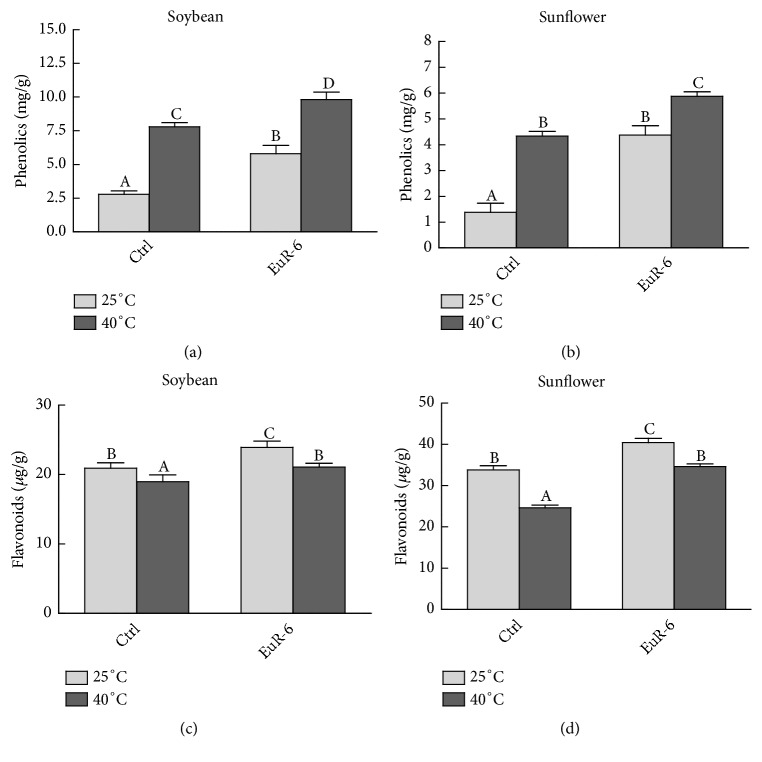
Effect of* A. flavus* on phenolics and flavonoids contents of soybean and sunflower seedlings. (a) represents the phenolics contents of soybean seedlings at 25°C and 40°C; (b) represents phenolics contents of sunflower seedlings at 25°C and 40°C; (c) represents flavonoids contents of soybean seedlings at 25°C and 40°C; (d) represents flavonoids contents of sunflower seedlings at 25°C and 40°C; Ctrl =* A. flavus*-free seedlings; EuR-6 =* A. flavus*-associated seedlings. Data are means of 3 replicates with standard error. Different litters are significantly different (p < 0.05) as estimated by Duncan's Multiple Range Test.

Total phenols, proline, and flavonoids content of soybean and sunflower seedlings maintained at 25°C or 40°C is presented in Figure 7. A significantly (P = 0.05) higher concentration of phenolics was founded in endophyte-associated seedlings of soybean and sunflower as compared to nonassociated seedlings grown at 25°C or 40°C (Figures 7(a) and 7(b)). Similarly, a significantly (P = 0.05) lower concentration of total flavonoids was detected in soybean and sunflower seedlings without* A. flavus* association and kept at 25°C or 40°C (Figures 7(c) and 7(d)).

### 3.7. Effect of* A. flavus* on CAT and AAO Activity of Soybean and Sunflower

**Figure 8:**
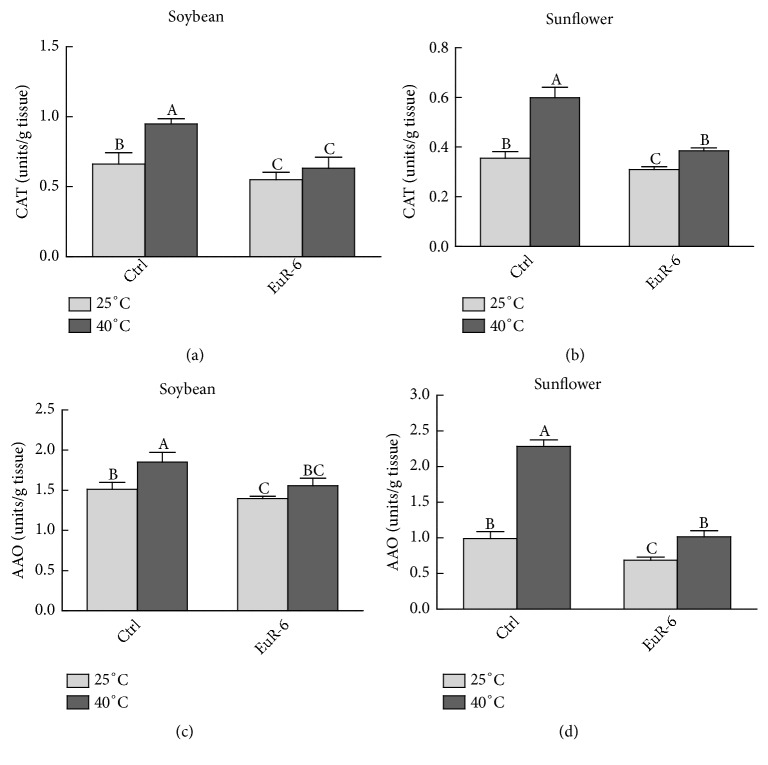
Effect of* A. flavus* on CAT and AAO activity of soybean and sunflower seedlings. (a) represents the CAT activity of soybean seedlings at 25°C and 40°C; (b) represents CAT activity of sunflower seedlings at 25°C and 40°C; (c) represents AAO activity of soybean seedlings at 25°C and 40°C; (d) represents AAO activity of sunflower seedlings at 25°C and 40°C; CAT = catalase; AAO = ascorbic acid oxidase; Ctrl =* A. flavus*-free seedlings; EuR-6 =* A. flavus*-associated seedlings. Data are means of 3 replicates with standard error. Different litters are significantly different (p < 0.05) as estimated by Duncan's Multiple Range Test.

A significant reduction was detected in the concentration of CAT and AAO in soybean and sunflower seedlings inoculated with* A. flavus* as compared to the control seedlings (Figure 8).* A. flavus*-associated soybean had significantly (P = 0.05) lower CAT as compared to the nonassociated soybean at both 25°C and 40°C (Figure 8(a)). Similarly, significantly (P = 0.05) higher activity of the CAT was noticed in the noninoculated sunflower seedlings in comparison to the inoculated seedlings at 25°C and 40°C (Figure 8(b)). Furthermore, a similar trend was noticed concerning the amount of AAO in soybean and sunflower seedlings associated with* A. flavus *at 25°C and 40°C as compared to* A. flavus*-free soybean and sunflower (Figures 8(c) and 8(d)).

### 3.8. Effect of* A. flavus* Inoculation on the Nutritive Value of Soybean and Sunflower

**Figure 9:**
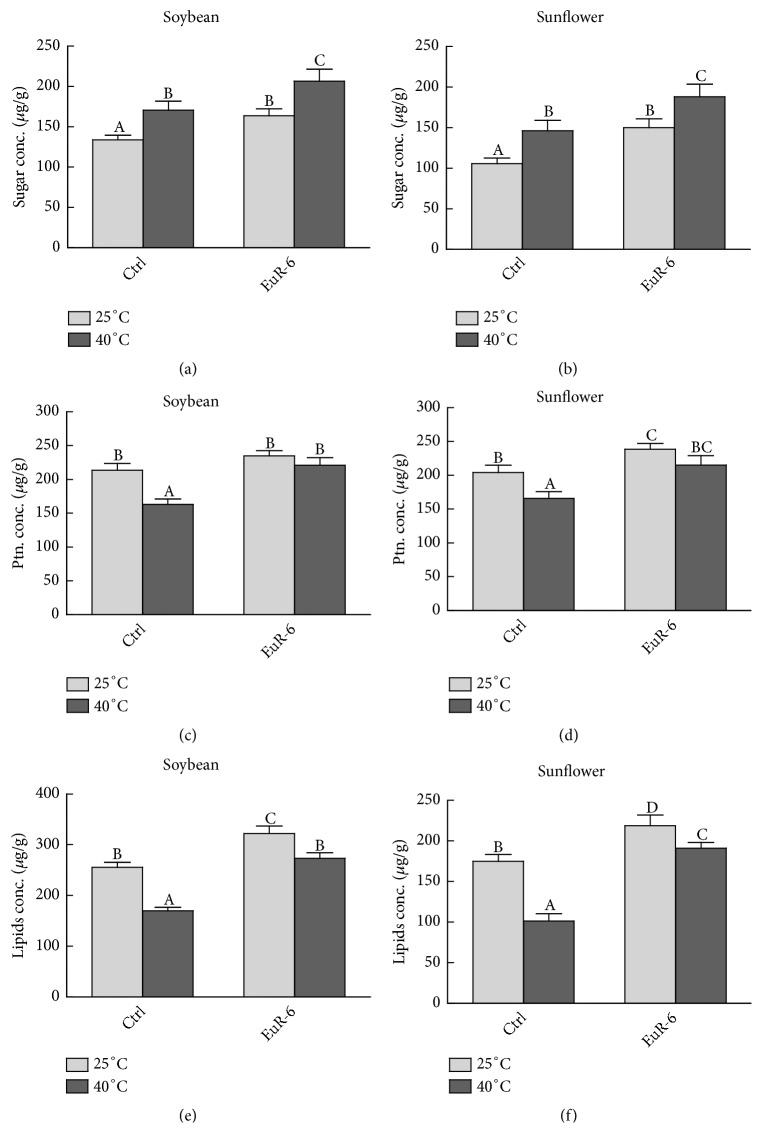
Effect of* A. flavus* on nutritional value of soybean and sunflower seedlings. (a) represents the total sugar concentration of soybean seedlings at 25°C and 40°C; (b) represents total sugar concentration of sunflower seedlings at 25°C and 40°C; (c) represents total protein concentration of soybean seedlings at 25°C and 40°C; (d) represents total protein concentration of sunflower seedlings at 25°C and 40°C; (e) represents lipids concentration of soybean seedlings at 25°C and 40°C; (f) represents lipid concentration of sunflower seedlings at 25°C and 40°C; Ptn. = protein; Ctrl =* A. flavus*-free seedlings; EuR-6 =* A. flavus*-associated seedlings. Data are means of 3 replicates with standard error. Different litters are significantly different (p < 0.05) as estimated by Duncan's Multiple Range Test.

*A. flavus-*associated soybean and sunflower seedlings had significantly (P = 0.05) higher amounts of soluble sugars at both 25°C and 40°C as compared to their respective controls (Figures 9(a) and 9(b)). Also, a significant increase was detected in the total protein contents of soybean and sunflower seedlings inoculated with* A. flavus *as compared to the soybean and sunflower controls at 25°C and 40°C (Figures 9(c) and 9(d)). A similar trend has been noticed for total lipids in* A. flavus*-associated soybean and sunflower seedlings in comparison to* A. flavus-*free soybean and sunflower at 25°C and 40°C (Figures 9(e) and 9(f)).

## 4. Discussion

Soybean and sunflower are one of the important oil and protein producing plants cultivated all over the world. But presently, these plants are facing many stress-challenges because of climate change. It is predicted by the global-climate-change analysis (GCCA) that 1–3.7°C rise in mean atmospheric temperature is expected at the end of the 21^st^ century [37]. Different techniques have been adopted by the researchers to enhance plant potential to cope with climate changes, including high temperature stress. Endophytes residing in the plant tissues can be one of the best options to mitigate heat stress. The endophytes can enable the host plants to produce potent bioactive compounds, like IAA, GA, flavonoids, phenolics, etc. during stress conditions to combat the threat [4–6]. Besides, endophytes also exhibit plant growth promoting activity that helps the host plant to stand biotic and abiotic stresses [7, 8]. In this regard,* A. flavus* also proved to promote soybean and sunflower growth under high temperature stress (40°C). Initial screening was carried out using rice seedlings due to quick response to GA and IAA, present in the culture filtrate of endophytic fungi [38]. Like many endophytic fungi,* A. flavus* also has the potential to secrete plant growth promoting IAA and mitigate heat stress by releasing phenols and flavonoids. In past,* Aspergillus niger, Paecilomyces formosus, Fusarium oxysporum, Rhizopus stolonifer, Penicillium funiculosum,* and* P. corylophilum* were found to release IAA and GA to support host plant growth [39, 40]. Plant-endophyte association is a type of mutualistic symbiosis during which endophytic partner secretes useful-secondary metabolites to support host growth [4, 6, 8]. Moreover, a high proline concentration was detected in endophyte-free soybean and sunflower at 40°C as compared to endophyte-associated soybean and sunflower. In a similar study, higher contents of proline were detected in tall fescue and rye grass under water stress [41]. Reduction in proline content in endophyte-associated seedlings at stress conditions suggests that endophytic fungi might convert proline into a by-product, such as loline alkaloids [42]. Like proline, ABA can act as a signaling agent during biotic and abiotic stresses. ABA has been noted to escalate in rice seedlings during high temperature stress [43]. This means that high temperature stress can upregulate the genes that are responsible for the ABA biosynthesis [44]. Low levels of ABA in* A. flavus*-associated soybean and sunflower seedlings maintained at higher temperature as compared to the* A. flavus*-free seedlings. This indicates the capacity of A*. flavus* in promoting the resistance in host seedlings against high temperature stress.

Also, due to unfamiliar environmental changes, plants produce higher quantities of phenolics to protect itself [45]. Various phenolics are accrued by higher plants during biotic and abiotic stresses to undergo normal growth [46]. In our study, the accumulation of higher quantities of phenolics in soybean and sunflower seedling during high temperature stress greatly supports the findings of Lattanzio et al. [47]. Similarly, an increase in flavonoid contents has been noticed in* A. flavus* inoculated soybean and sunflower seedlings, which suggested the positive role of* A. flavus* in improving the nutritional value of both species. The results further confirmed the findings of Yang, Ma, Yuan, Huang, Yang, Zhang, Huang, Ren, and Shan [48], who reported higher flavonoids in fungal-associated wine grapes. Besides the normal growth promotion,* A. flavus* has better the nutritional quality of both soybean and sunflower under stress condition. The* A. flavus* inoculated soybean and sunflower seedlings had higher amounts of soluble sugars, total proteins and total lipids as compared to the* A. flavus*-free seedlings of both species. Similar observations have been noted by Clifton, Jaronski, Coates, Hodgson and Gassmann [49], who said that the elevation in nutritional quality of soybean was due to fungi,* M. brunneum*. It might be possible that endophytes might help the host plants to absorb optimum amounts of nutrients from rhizosphere to fulfill their nutrient requirements even under stress conditions.

All plants generate ROS, which are highly unstable free radicals having a lone pair of electrons. Low concentration of ROS in cells is very essential for the healthy growth of plants as they help in cell division, progression, differentiation, intracellular signaling, and defense against pathogens. However, higher concentrations of ROS may disrupt the important physiological processes and leads to enhanced aging and apoptosis [50]. In fact, all living organisms have a special antioxidant defense system (ADS) that stabilize or deactivate ROS by interrupting their oxidizing chain reactions before harming the cells [51]. Plants have a potential source of the enzymatic antioxidant defense system, including catalase and ascorbic acid oxidase [52]. In plants, excess of H_2_O_2_ is produced in response to biotic and abiotic stresses. The production of higher amounts of H_2_O_2_ can cause oxidative damage to important cell components. Catalase and ascorbic acid oxidase can ease the stress by degrading the excess ROS, i.e., H_2_O_2_, singlet oxygen, superoxides, and hydroxyl radicals [53]. Low level of catalase and AAO in soybean and sunflower seedlings associated with* A. flavus* at high temperature (40°C) clearly indicates its role in stress resistance. This study suggests that endophytic fungus* A. flavus* plays significant role in the lowering high temperature stress as previously reported by Hamayun, Hussain, Khan, Kim, Khan, Waqas, Irshad, Iqbal, Rehman, and Jan [4]. Certainly,* A. flavus* helped the host plants to ameliorate both biotic and abiotic stresses that may be a new gateway to neutralize the consequences of global warming faced by agricultural crops.

## 5. Conclusion

High temperature is one of the potent hazardous abiotic stresses to food crops, causing reduction in their quality and quantity. Present work on endophytic fungus* A. flavus* proved its capability to mitigate thermal stress in soybean and sunflower seedlings.* A. flavus* has enhanced the growth while reduced the phenol, catalase, and ascorbic acid oxidase contents in associated seedlings under high temperature stress. The fungus also secreted vital secondary metabolites (IAA, SA, phenol and flavonoids) that supported the growth of the host plant species. So, it is vital to use economical, long lasting, and eco-friendly natural resources, for, e.g.,* A. flavus* to promote host plant growth under heat stress.

## Data Availability

The data used to support the findings of this study are included within the article.

## Supplementary Materials

Supplementary Materials
* Supplementary 1*.* Figure S1:* colonies of EuR-6 grown on Hagem minimal medium and purified on PDA media plates, isolated from* Euphorbia indica *L. EuR represents that the strain was isolated from the* Euphorbia indica *L. root.* Supplementary 2*.* Figure S2:* preliminary screening of* Aspergillus violaceofuscus* (DryL-1) filtrate (100 *μ*l) isolated from* Dryopteris* L. on rice seedlings at 2 leaves stage grown in 0.8% water-agar medium for 2 weeks at 25°C. Reading taken after 1 week of culture filtrate application. 10 sets of pots. Each set has 3 treatments, including Czapek control (right), distilled water control (left), and endophyte cultural filtrate (middle).
