# Supplementary material for: Aspergillus flavus Promoted the Growth of Soybean and Sunflower Seedlings at Elevated Temperature
Source: Biomed Res Int. 2019 May 2;2019:1295457. doi: 10.1155/2019/1295457 (PMC6521304; doi:10.1155/2019/1295457)
Supplement: Supplementary Materials — Supplementary 1. Figure S1: colonies of EuR-6 grown on Hagem minimal medium and purified on PDA media plates, isolated from Euphorbia indica L. EuR represents that the strain was isolated from the Euphorbia indica L. root. Supplementary 2. Figure S2: preliminary screening of Aspergillus violaceofuscus (DryL-1) filtrate (100 μl) isolated from Dryopteris L. on rice seedlings at 2 leaves stage grown in 0.8% water-agar medium for 2 weeks at 25°C. Reading taken after 1 week of culture filtrate application. 10 sets of pots. Each set has 3 treatments, including Czapek control (right), distilled water control (left), and endophyte cultural filtrate (middle). [file 1295457.f1.docx]

**
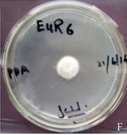
**

**Figure S1:** Colonies of EuR-6 grown on Hagem minimal medium and purified on PDA media plates, isolated from *Euphorbia indica* L. EuR represents that the starin was isolated from the *Euphorbia indica* L. root.

**
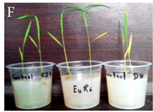
**

**Figure S2:** Screening bioassay of EuR-6 (100μl) isolated from *Euphorbia indica* L. on rice seedlings at 2 leaves stage grown in 0.8% water-agar medium for 2 weeks at 25^o^C. Reading taken after 1 week of culture filtrate application. Czapek control (right), distilled water control (left) and endophyte cultural filtrate (middle).
